# Turkish Baths

**Published:** 1877-04-20

**Authors:** 


TURKISH BATHS.—As matter of in formation and interest to any of our readers who may not be acquainted with the history and processes of the famous Turkish Bath, we publish an article on the subject in the present issue, by one, who, having long had the bath in successful operation in this city, is practically familiar with its details and effects.

WE INVITE attention to the advertisement of Geo. J. Howard, Druggist. Mr. Howard is a man of high integrity, and of well established business reputation in this city. We can with confidence and pleasure recommend his House to the patronage of our readers.

